# Effects of postharvest collision damage on qualities of kiwifruit during storage

**DOI:** 10.3389/fpls.2025.1683638

**Published:** 2025-10-02

**Authors:** Ke He, Mengmeng Qiao, Wenzheng Liu, Xiangyu Sun, Yulin Fang, Yuan Su

**Affiliations:** ^1^ College of Mechanical and Electronic Engineering, Northwest A&F University, Yangling, China; ^2^ College of Mechanical and Electronic Engineering, Nanjing Forestry University, Nanjing, China; ^3^ College of Enology, Northwest A&F University, Yangling, China

**Keywords:** kiwifruit, repeated collision, fruit surface zone, physicochemical properties, storage

## Abstract

**Introduction:**

Understanding the physicochemical quality variations of kiwifruit subjected to repeated collisions during storage is critical for optimizing postharvest processing and improving handling equipment. However, this issue has received limited research attention. This study investigated the effects of different factors (storage time, collision position, and collision frequency) on the quality of kiwifruit during storage. This study provides new insights into the preservation and storage of kiwifruit.

**Methods:**

In this study, the effects of three collision frequencies (1, 3, and 5 impacts), three collision positions (top/stem shoulder, middle/cheek, and bottom/calyx shoulder) simulated using an impactor, and a storage period of 42 days were evaluated on the physicochemical properties of kiwifruit. Multivariate analysis of variance was performed on weight loss rate (WL), hardness, soluble solids content (SSC), titratable acidity (TA), reducing sugars (RS), and vitamin C (VC).

**Results:**

The results showed that these factors significantly influenced WL and the hardness of kiwifruit. The collision frequency and collision position both affected SSC and RS, whereas collision frequency significantly influenced VC content but not TA. On the contrary, the collision position had no significant effect on VC content but significantly affected TA. The coefficient of determination R2 for all multiple regression models exceeded 0.5. Furthermore, correlation analysis demonstrated that repeated collisions accelerated kiwifruit ripening, and weakened the correlation among physicochemical properties.

**Discussion:**

Overall, this study highlights the substantial impact of mechanical damage on the physicochemical quality attributes of kiwifruit during storage, offering a new perspective for assessing damage sensitivity under different storage conditions.

## 1 Introduction

Kiwifruit, a fruit highly prized for its rich nutritional profile, is particularly noted for its high Vitamin C content, dietary fiber, and antioxidant properties (Harker et al., 2019; Liang et al., 2022). The fruit is widely consumed globally, both in its fresh form and as an ingredient in various food products (Bassi et al., 2023). Postharvest losses of kiwifruit occur throughout multiple different stages of the supply chain, including harvesting, storing, handling, packaging, transporting, and marketing (Al-Dairi et al., 2022). During these processes, fruit may be bruised through mechanical stress, including fingerprints, dropping, squeezing, and packing pressure (Wang et al., 2021; Zhu et al., 2022). Collision damage represents one of the most critical quality challenges during transportation. This mechanical damage can result in bruising and severely reduce marketability by altering the fruit’s physical and chemical properties. Research indicates that mechanical damage can lead to substantial postharvest losses ranging from 30% ~ 50% (Du et al., 2019). Consequently, comprehending the effects of collision damage on kiwifruit qualities is essential for devising strategies to mitigate postharvest losses and prolong shelf life.

During mass harvesting using a shake-and-catch method or postharvest handling (e.g., transportation), fruit-to-fruit or fruit-to-limb are generally unavoidable (Li et al., 2023; Zaharan et al., 2020). These impact forces are typically low in magnitude and may not leave visible damage on the fruit surface. However, such mechanical impacts can negatively affect fruit quality during storage. Mechanical damage resulting from impact forces is a major contributor to the deterioration of overall quality attributes and the economic value of produce. Fu et al. (2023) employed the pendulum method to measure bruising in apples caused by repeated low-intensity collisions. They found that repeated collisions with lower intensity were preferable to reduce bruise damage. The zone near the fruit stem was more susceptible to causing smaller bruise sizes. Similarly, Wang et al. (2019) investigated the impact behavior and damage level in litchi and found that most damage occurred after ten fruit-to-fruit collisions. Accordingly, it was hypothesized that different zones on the kiwifruit surface would bear varying amounts and susceptibility of bruising, depending on the locations of the kiwifruit surface and collision frequency. Understanding the physicochemical properties change of kiwifruit to bruising during storage after multiple impacts is essential for developing strategies to minimize postharvest losses.

Numerous studies have examined the effects of various storage conditions on kiwifruit qualities, with particular attention to factors such as variety, loading methods, storage temperature, humidity, and the application of preservatives (Zolfaghari et al., 2010; Ban et al., 2024; Chai et al., 2019; Xu et al., 2021; Polychroniadou et al., 2022). Nalan and Nurdan (2024) investigated the relationship between harvest time and physicochemical quality characteristics of ‘Hayward’ cultivar fruit during cold storage, finding that the qualities of kiwifruit varied with harvest maturity under long-term refrigeration. Song et al. (2022) established a vibrational bruise prediction model based on a backpropagation (BP) neural network to assess bruise damage in harvested kiwifruit. Their findings indicated that vibration-induced damage and deformation could be predicted based on vibration acceleration, frequency, and duration. Overall, previous studies have focused on the quality changes in undamaged kiwifruit during storage or on the quantitative analysis of mechanical damage resulting from compression and impact forces.

The quality variations of kiwifruit damaged by collision during storage are different from those of undamaged kiwifruit. Existing research has primarily focused on the influence of storage conditions on fresh, intact kiwifruit and the quantitative analysis of damage severity. However, the effects of collision damage and the influence of kiwifruit quality during storage remain uninvestigated. The specific effects of collision damage on postharvest quality during storage remain largely unexplored. Therefore, this study investigates how repeated collisions at different surface locations affect the postharvest physicochemical properties of kiwifruit, using a pendulum impact test. Thus, the study aimed to: (1) investigate the effects of three factors including storage duration (42 days), and three collision positions and three collision frequency on the physicochemical quality attributes of kiwifruit, where few studies have been discussed and (2) examine the influence of the of these independent variables (storage duration, collision frequency and collision position) on the dependent variables, including weight loss (WL), hardness, soluble solids content (SSC), reducing sugars (RS), titratable acidity (TA), vitamin C (VC) content, (3) determine the correlations among physicochemical parameters during storage.

## 2 Materials and methods

### 2.1 Plant material and storage conditions

The ‘Xu Xiang’ kiwifruits were hand-harvested at the physiological maturity stage from an orchard located in Yangling, Xianyang, China (108.06°E, 34.26°N) on the same day. Fruits of uniform size and free from visual defects, including rot, splitting, shrivelling, absence of bloom (wax), and bruising, were selected for further analysis. Following the harvest, the fruits were transported to the laboratory at Northwest A&F University for impact treatment, as described in Section 2.2. The kiwifruit were subsequently stored in a temperature-controlled chamber at 4 °C and 72% relative humidity.

### 2.2 Experimental apparatus

**Figure 1:**
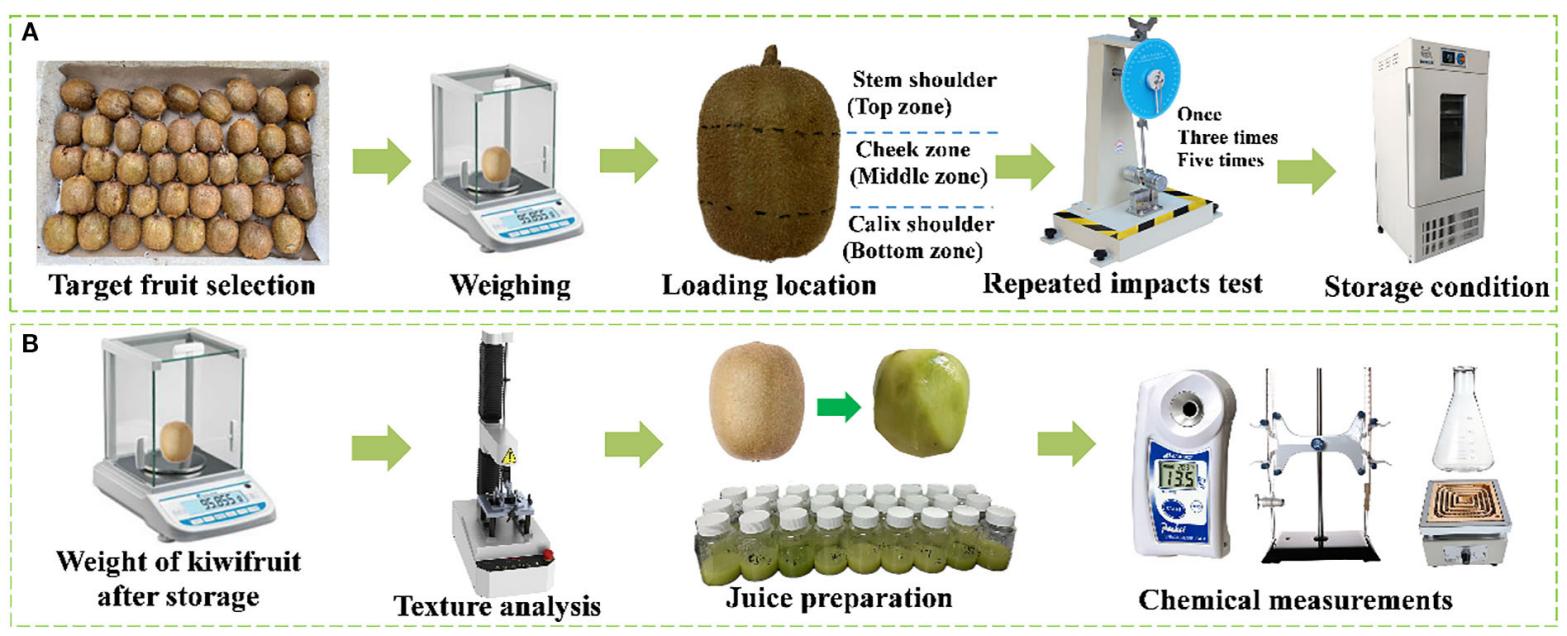
**(A)** Schematic of the pendulum-type impact device and experimental setup **(B)** Physical and chemical quality testing.

A series of repeated impact tests, conducted once, three times, and five times, were performed to evaluate effects on impact parameters and physicochemical properties, focusing on different surface zones of the kiwifruit: the stem shoulder, cheek area, and calyx shoulder, which are referred to as the top, middle, and bottom zones. The definitions of these three surface zones are illustrated in **Figure 1A**. Before the impact tests, the impact regions on the kiwifruits were outlined and marked with black pens. In contrast, undamaged kiwifruits served as the control group. The impact tests were carried out using a pendulum-type device consisting of a 400 mm long arm, a rotating angular indicator, an arm positioning mechanism, and a piezoelectric impact force sensor (JHBM-H1, Bengbu sensor system engineering Co., Ltd, China) equipped with a stainless-steel cylindrical impactor, all mounted on an aluminium frame. During testing, the pendulum arm was manually drawn back to a fixed position and released to strike the kiwifruit. The impact force and contact duration between the fruit and the impactor were recorded using an instrument information card (USB3100N, Aertai Corp., China) at a sampling rate of 20 kHz, and the data were processed using Art DQA software. The average impact force was measured at 2.94 ± 1.44 N, a magnitude consistent with forces typically encountered by kiwifruit during harvesting, packaging, and transportation (Chen et al., 2024).

Generally, the total number of treatments was 9 and each treatment included 5 replicates. The study lasted for 42 days, and the investigated physicochemical parameters were analyzed at intervals of 1, 3, 5, 7, 16, 21, and 42 days. To avoid the influence of low temperature on the testing of kiwifruit, all evaluations were conducted at 20 °C after 2 hours of removing the kiwifruit from the temperature-controlled chamber. The postharvest physicochemical properties were assessed in the following sequence: weight loss (WL), hardness, and selected chemical parameters, including reducing sugars (RS), soluble solids concentration (SSC), total acidity (TA) and vitamin C (VC). The testing procedure is illustrated in **Figure 1B**.

### 2.3 Physical attributes measurements

Each kiwifruit was weighed using a digital balance with a precision of 0.01 g (CN-LQ10002, Leqi, China). The weight loss rate is calculated using Equation 1.


(1) $$w=\frac{M_{0}-M_{1}}{M_{0}}\times 100$$

Where, *M_0_* and *M_1_* are the weight of kiwifruit before and after storage, respectively (g), and the *w* is the weight loss rate (%).

The same surface zone of the kiwifruit impacted by the pendulum-type device was first assessed for firmness using a P2 probe on a texture analyzer (TA.XT.Plus C, Stable Micro Systems, Inc., England) at a speed of 3 mm/s and a penetration depth of 5 mm (Chen et al., 2024). It was worth noting that when evaluating the hardness of kiwifruit, all the kiwifruit were peeled. The average value was measured as the reference for sample hardness. Hardness measurements for each kiwifruit were conducted in triplicate. Subsequently, fresh juice was extracted from each kiwifruit for chemical analysis.

### 2.4 Chemical attributes measurements

Kiwifruit juice was prepared through the processing of the flesh in a juicer, followed by the filtration of larger impurities using a filter cloth. The soluble solids content (SSC) of the juice was determined using a handheld refractometer (ATAGO PAL-1, model 8101, Japan) with juice extracted subsequent to texture analysis. Reducing sugar (RS) was determined according to the National Standard of the People’s Republic of China (GB/T 15038-2006, 2006). Titratable acidity (TA) was assessed through colorimetric titration using phenolphthalein and a 0.1 mol L⁻¹ NaOH solution, with results expressed as malic acid equivalents per 100 mL of kiwifruit juice, based on a malic acid standard curve. Vitamin C (VC) content was quantified as L-ascorbic acid and measured by colorimetric titration with 0.05 mol L⁻¹ 2,6-dichloroindophenol sodium (GB 14754-2010, 2011). Three replicate measurements for each group were conducted, with the mean taken as the experimental value. The initial properties of the selected kiwifruit are shown in **Table 1**.

**Table 1:** The initial characteristics of fresh kiwifruit selected for the study.

Main characteristics	Value
Weight (g)	114.89 ± 8.66
Firmness (N)	31.62 ± 5.80
SSC (%)	13.93 ± 0.83
RS (g/L)	10.47 ± 0.24
TA (g/L)	6.36 ± 0.21
VC (mg/100g)	56.5 ± 0.26

### 2.5 Pearson correlation and multiple regression analysis

For Pearson’s correlation coefficient (r) (Equation 2), heat map charts were utilized to graphically show the correlation matrices between the quality attributes among all tested conditions. Each cell in the correlation matrix corresponds to the correlation coefficient (r). The color range of the heat map cells from green (high correlation, *r* = 1) to red (high anti-correlation, *r* = -1), passing from light yellow (no correlation, *r* = 0). The significance level was set to α = 0.001.


(2) $$r=1-\frac{n(\sum xy)-(\sum x)(\sum y)}{\sqrt{\left[n\sum x^{2}-{\left(\sum x\right)}^{2}][n\sum y^{2}-{\left(\sum y\right)}^{2}\right]}}$$

Where, *n* is the number of data points, i.e., (*x, y*) pairs, in the data set.

Also, the multiple regression model was applied to investigate the influence of independent variables (storage duration, collision times, and collision position) on the dependent variables (weight loss, hardness, soluble solids content, reducing sugar, titratable acidity, and VC) at a 5% significance level. Besides, the determination coefficient (R^2^), maximum and minimum residuals, and f-test were recorded to determine the accuracy of each model.

### 2.6 Statistical analysis

IBM SPSS Statistics 26.0 (International Business Machine Crop., New York, USA) was used for statistical analysis. The multivariate analysis of variance was applied to investigate the effects of collision frequency (once, three times, and five times), collision position (top zone, middle zone, and bottom zone), and storage duration on the physical quality (WL and hardness) and chemical attributes (SSC, RS, TA and VC) at a 95% significance level. Comparisons of physical and chemical attributes were performed using the least significant difference (LSD) *post hoc* test at α = 0.05. Graphs were generated using Origin 2021b software (OriginLab Corp., Northampton, MA, USA). All results are presented as mean ± standard deviation.

## 3 Results and discussion

### 3.1 Effect of storage duration, collision frequency, and collision positions on the physical attributes

#### 3.1.1 Weight loss

**Figure 2:**
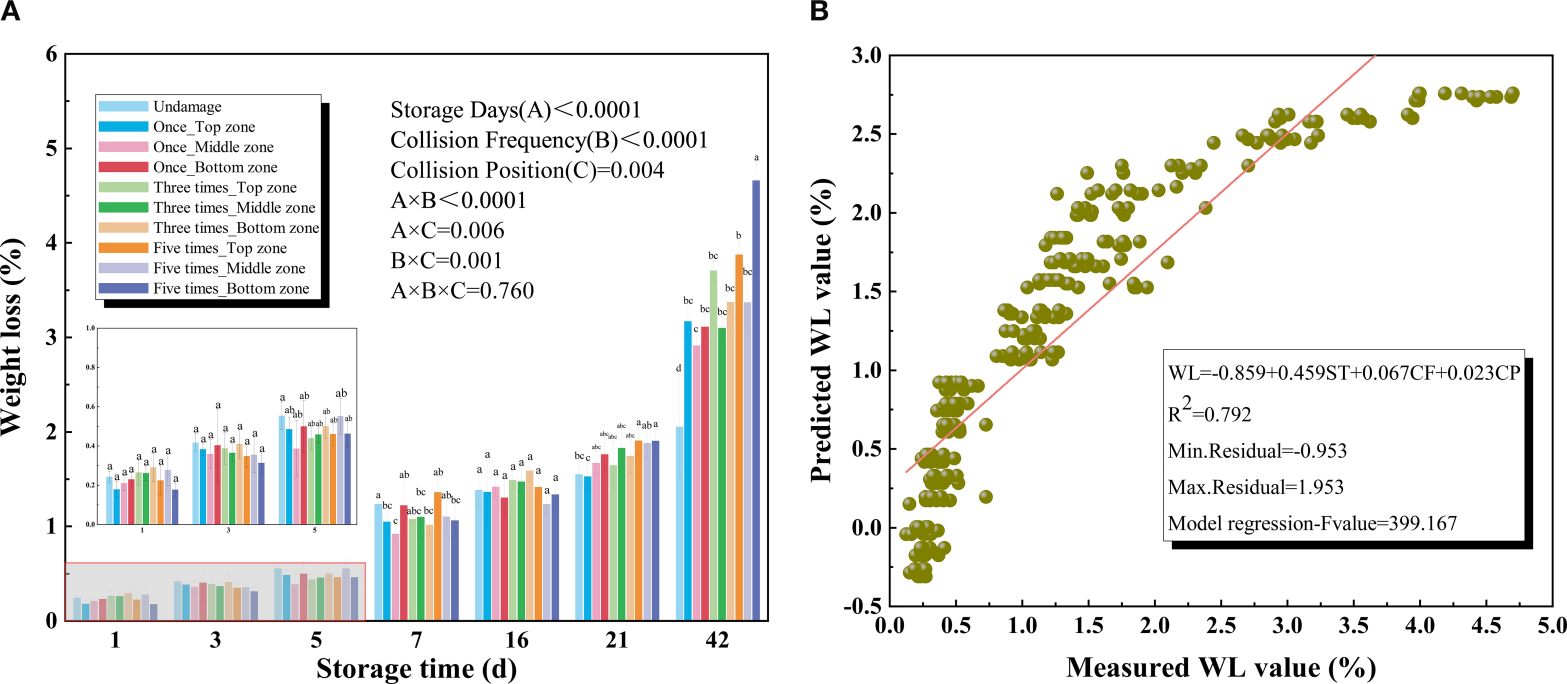
**(A)** Weight loss (WL) of different collision positions and frequency over 42 days **(B)** Results for the prediction models of weight loss (WL) based on multiple regression models.

**Figure 2A** showed that under different collision conditions, the WL of kiwifruit increased significantly with prolonged storage. During the early stage of storage, WL remained relatively low, whereas in later stages, the variability in WL became more pronounced. The storage duration had a significant effect on WL (P < 0.0001). Within the first 21 days of storage, repeated collisions had no significant effect on WL; however, WL gradually increased with higher collision frequencies, and this effect intensified with longer storage duration. Especially, on the 42nd day, the average WL of the kiwifruit bottom zone after one impact, three impacts, and five impacts was 3.11%, 3.37% and 4.65%. Thereby, the collision frequency had a significant effect on WL (P < 0.0001). The cheek zone of kiwifruit exhibited the greatest resistance to mechanical damage compared with other positions, indicating the collision position significantly influenced WL during storage (P = 0.004).

In the early storage stage, kiwifruit retained most of its moisture and mechanical damage exerted only a limited effect. However, in the later stage of storage, the weight loss of damaged kiwifruit was significantly higher than that of undamaged kiwifruit. This is because the impact load causes intracellular damage to the fruit tissue cells, accelerating water loss and withering processes (Wei et al., 2019). Notably, in later stages, variability in WL was observed to increase, likely due to the cumulative effects of repeated collisions and the progressive weakening of the fruit structure over time (Islam et al., 2012). This variability may also be influenced by cultivar, maturity, and skin condition, which affect the balance among evaporation, oxidation, and microbial activity (Rivera et al., 2021; Ali et al., 2024). The cheek zone of kiwifruit exhibits the highest resistance to mechanical damage, potentially due to its thicker peel, densely arranged cells, and lower curvature. These characteristics facilitate more uniform stress distribution upon impact, thereby minimizing localized cell rupture. Consequently, post-impact increases in WL remained relatively moderate. **Figure 2B** presents the final regression model, with storage duration, collision frequency, and collision position as independent variables. The comparison between predicted and measured WL indicated a good fit (R² = 0.792).

#### 3.1.2 Hardness

**Figure 3:**
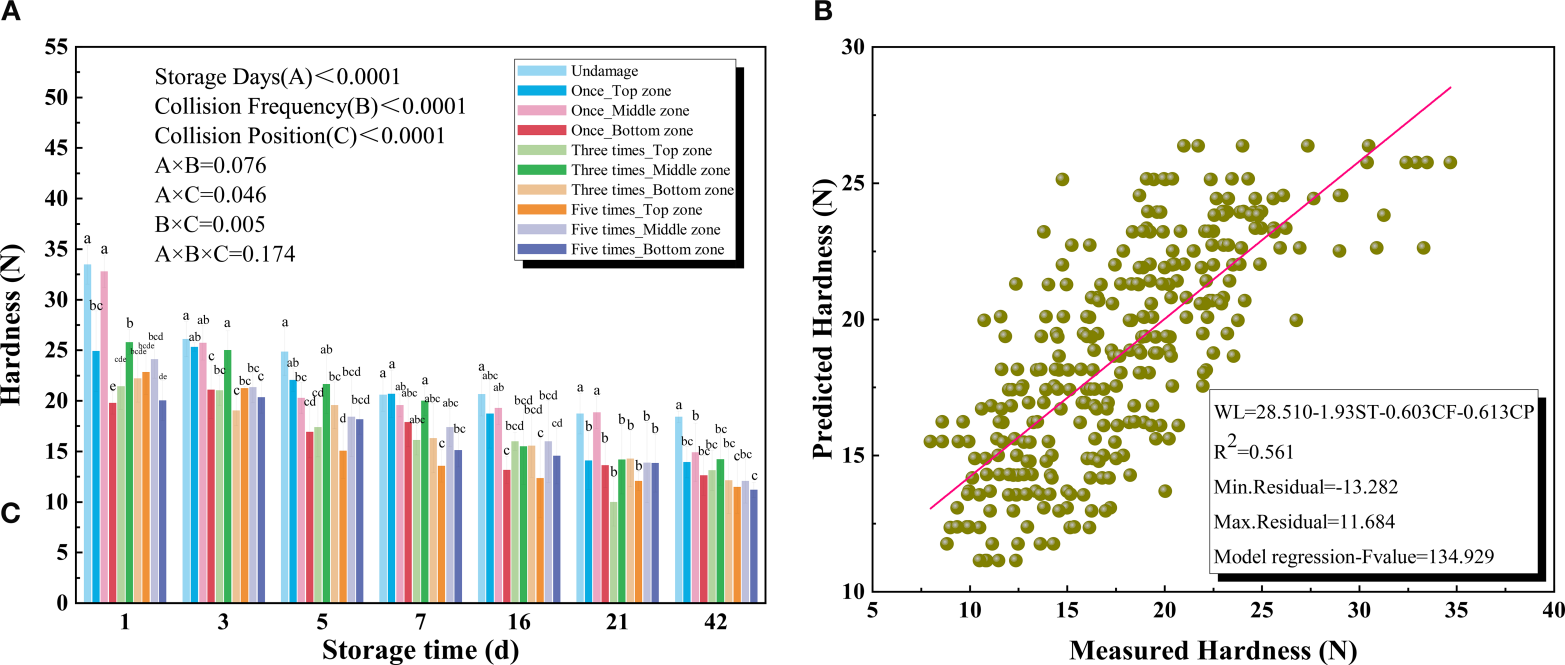
**(A)** Hardness of different collision positions and frequency over 42 days **(B)** Results for the prediction models of hardness based on multiple regression models.

As shown in **Figure 3A**, kiwifruit hardness consistently decreased throughout storage. The maximum hardness was 33.46 N on Day 1, while the minimum hardness was 13.56 N on Day 7, indicating pronounced softening during the early storage stage. With prolonged storage, hardness further decreased to 11.19 N on Day 42. These results indicated that storage duration had a significant effect on the hardness reduction of kiwifruit during storage (P<0.0001). The repeated impacts significantly influenced the rate of hardness reduction, with multiple impacts accelerating softening (P<0.0001). **Figure 3A** also showed that the collision position significantly affected kiwifruit hardness during storage (P < 0.0001).

Changes in hardness generally reflect kiwifruit maturity and are important indicators of fruit quality (Li et al., 2024). On the one hand, mechanical damage can disrupt cell walls in the flesh, promote microbial infection, and significantly accelerate fruit softening (Sharma et al., 2009). On the other hand, when the cell structure of kiwifruit is disrupted, both respiration and transpiration rates increase, leading to accelerated loss of water and nutrients (Xanthopoulos et al., 2017). The degradation of cell wall components such as cellulose and pectin is exacerbated by mechanical damage, which weakens the intercellular connections and contributes to fruit softening (i.e., decreased firmness). Similar findings were reported by Pan et al. (2022) in their study on cell wall metabolism in small white apricots subjected to low-temperature plasma treatment. Consequently, repeated collisions can rapidly decrease kiwifruit hardness. Compared with other surface areas, the kiwifruit stem shoulder exhibited greater resilience to impact stress, whereas the calyx shoulder softened the fastest after impact. This may be attributed to the larger curvature of the calyx shoulder, which increases stress concentration during impact (Mao et al., 2023; Ban et al., 2024). Therefore, avoiding repeated collisions on the calyx shoulder of kiwifruit is essential for maintaining firmness during storage. **Figure 3B** presented the hardness regression model with all independent variables. The coefficient of determination (R^2^) between the predicted and measured values of hardness was 0.561.

### 3.2 Effect of storage duration, collision frequency, and collision positions on the chemical index

#### 3.2.1 Soluble solids content

**Figure 4:**
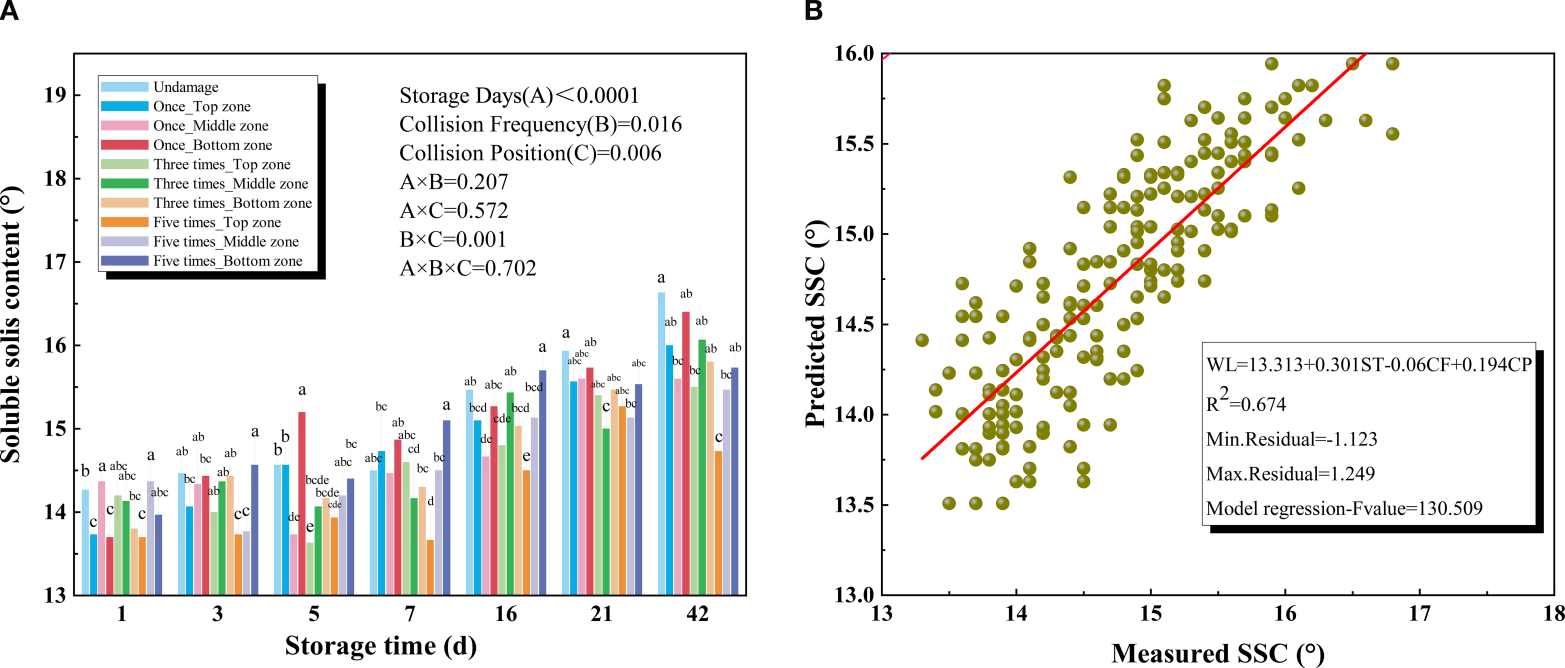
**(A)** Soluble solids content (SSC) of different collision positions and frequency over 42 days **(B)** Results for the prediction models of soluble solids content (SSC) based on multiple regression models.

SSC is a crucial indicator of postharvest quality in kiwifruit. During the experimental storage period, SSC ranged from a maximum value of 14.36% on day 1 to a maximum value of 16.63% on day 42, showing a significant upward trend with prolonged storage. The increase was more pronounced during the first week than in later stages, consistent with the findings of Pathare and Al-Dairi (2021). As shown in **Figure 4A**, SSC varied slightly with collision frequency and collision position. The analysis of variance revealed the significant relationship between SSC and collision frequency (P = 0.016) and collision position (P = 0.006). Compared to the other two collision positions, the stem shoulder of kiwifruit exhibited the greatest sensitivity to impact, significantly influencing SSC.

Regarding collision damage, Chai et al. (2019) observed that SSC increased with rising storage temperature and duration during kiwifruit ripening. Hussein et al. (2020) found no significant relationship between SSC and impact bruising in pomegranates. However, Maia et al. (2011) reported an increase in SSC in bananas following bruise damage, which was attributed to the conversion of starch to soluble sugars during the ripening process (Al-Dairi et al., 2021; Gao et al., 2021). Mechanical injury can induce stress responses in the fruit, enhancing the activity of ripening-related enzymes (e.g., pectinase, amylase), which further promotes cell wall degradation and nutrient transformation. This includes the accelerated conversion of starch to soluble sugars, thereby affecting SSC (Polychroniadou et al., 2022). **Figure 4B** presented the results of the SSC model and all independent variables. The coefficient of determination (R2) was 0.674 between the predicted and measured values of SSC.

#### 3.2.2 Reducing sugar

**Figure 5:**
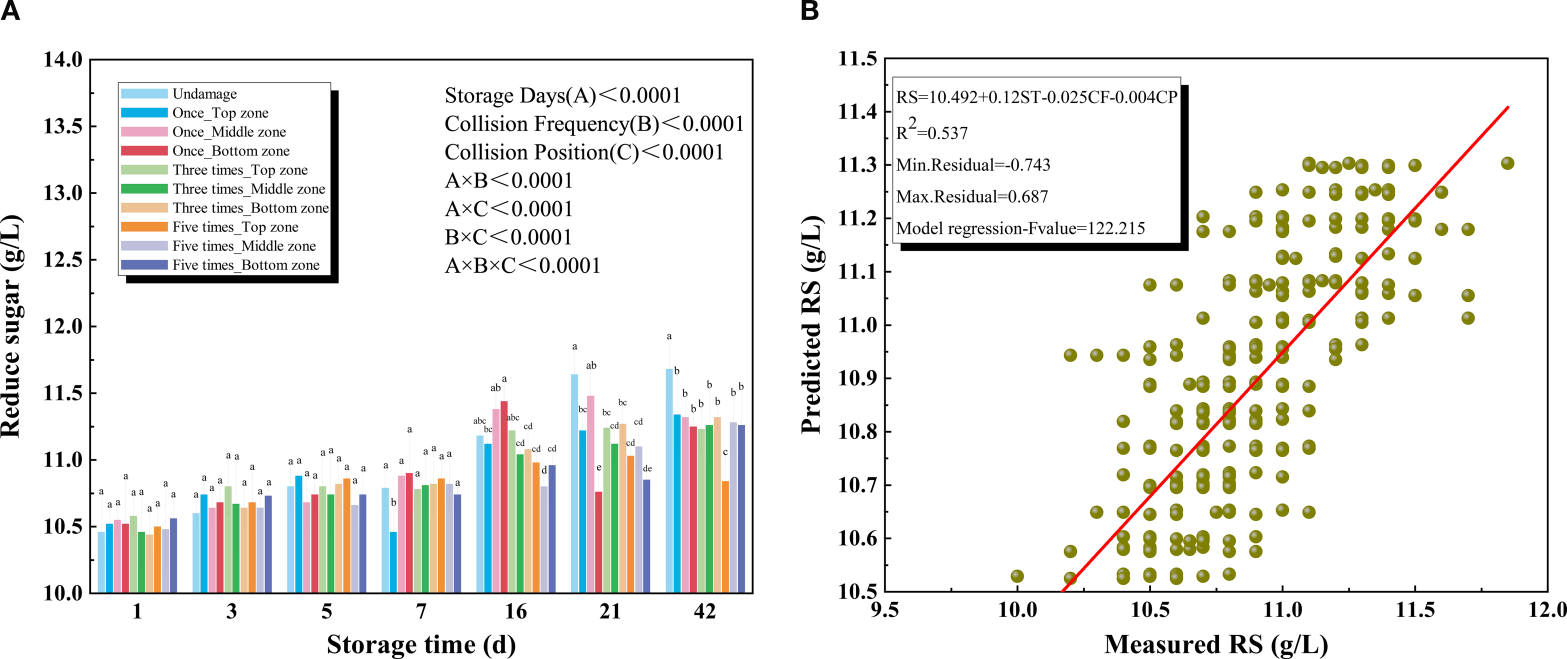
**(A)** Reducing sugar (RS) of different collision positions and frequency over 42 days **(B)** Results for the prediction models of soluble reducing sugar (RS) based on multiple regression models.

As shown in **Figure 5A**, the RS content of kiwifruit increased significantly with prolonged storage. The rate of increase was greater during the first week than in later stages. After 42 days of storage, the RS content of kiwifruit reached 11.30 g/L. This trend can be attributed to the enzymatic conversion of starch into reducing sugars primarily glucose and fructose, leading to their gradual accumulation during storage (Yu et al., 2024). Analysis of variance demonstrated that storage duration, collision frequency, and collision position all exerted significant effects on RS (P < 0.001). However, during the early storage period, no significant differences were observed among different collision treatments. Significant variation in RS content emerged only when the top zone was impacted, with differences becoming apparent after 42 days of storage under varying impact frequencies. Overall, undamaged kiwifruits consistently exhibited higher RS content than those subjected to mechanical damage, with the disparity becoming more pronounced during the later stages of storage under repeated impacts. Among the three collision positions, impacts on the cheek region exerted the least influence on RS content.

Kiwifruit subjected to mild impact damage during short-term storage (within two weeks) may compensate for metabolic disturbances through intrinsic self-repair mechanisms, thereby exhibiting no significant RS differences. A similar observation was reported by Pathare et al. (2023) in their study on simulated impact effects in stored pomegranates. In contrast, during long-term storage (beyond three weeks, particularly after six weeks), cumulative damage exceeds the fruit’s capacity, leading to progressive cellular degradation and intensified metabolic disorders. This process results in significant differences in response variables related to collision frequency and position (Ren et al., 2020). As shown in **Figure 5B**, the RS prediction model yielded a coefficient of determination (R²) of 0.537 between predicted and observed values.

#### 3.2.3 Titratable acidity

**Figure 6:**
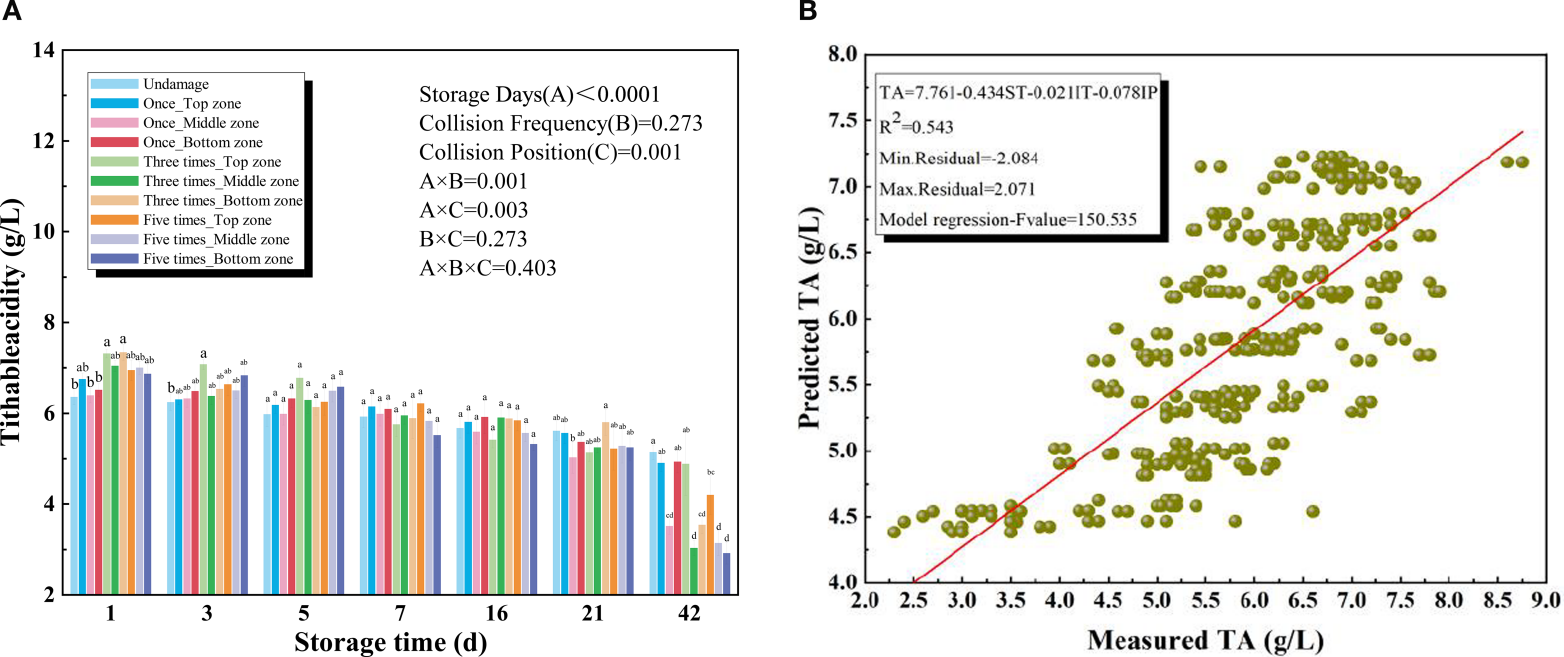
**(A)** Titratable acidity (TA) of different collision positions and frequency over 42 days **(B)** Results for the prediction models of soluble titratable acidity (TA) based on multiple regression models.

As shown in **Figure 6A**, the TA of kiwifruit significantly decreased with the increase of storage period. The analysis of variance showed no relation between the collision frequency and the TA value of the kiwifruit (P = 0.273). On the contrary, the collision position exerted a significant influence (P = 0.001). When the calyx shoulder of the kiwifruit was subjected to one or repeated collisions, no significant differences in TA were observed between impacted and non-impacted fruit during the early storage period. However, by the end of six weeks, the lowest TA value (2.94 g/L) was recorded in kiwifruits subjected to five impacts at the calyx shoulder.

The reduction of TA is associated with accelerated fruit senescence, as noted by Shahkoomahally and Ramezanian (2015). The overall decrease in acidity during storage has been attributed to the respiratory consumption of organic acids in fresh fruit (Mubarok et al., 2022). Similarly, Nayab and Akhtar (2023) reported a marked decline in TA in bananas, which became inedible after 28 days of storage, likely due to dynamic changes in malic, oxalic, and citric acid levels during the onset of ripening. Therefore, over the entire storage period, the TA of kiwifruit exhibits a continuous decline trend. **Figure 6B** presented the results of the TA model and all independent variables. The coefficient of determination (R^2^) was 0.543 between the predicted and measured values of TA.

#### 3.2.4 Vitamin C

**Figure 7:**
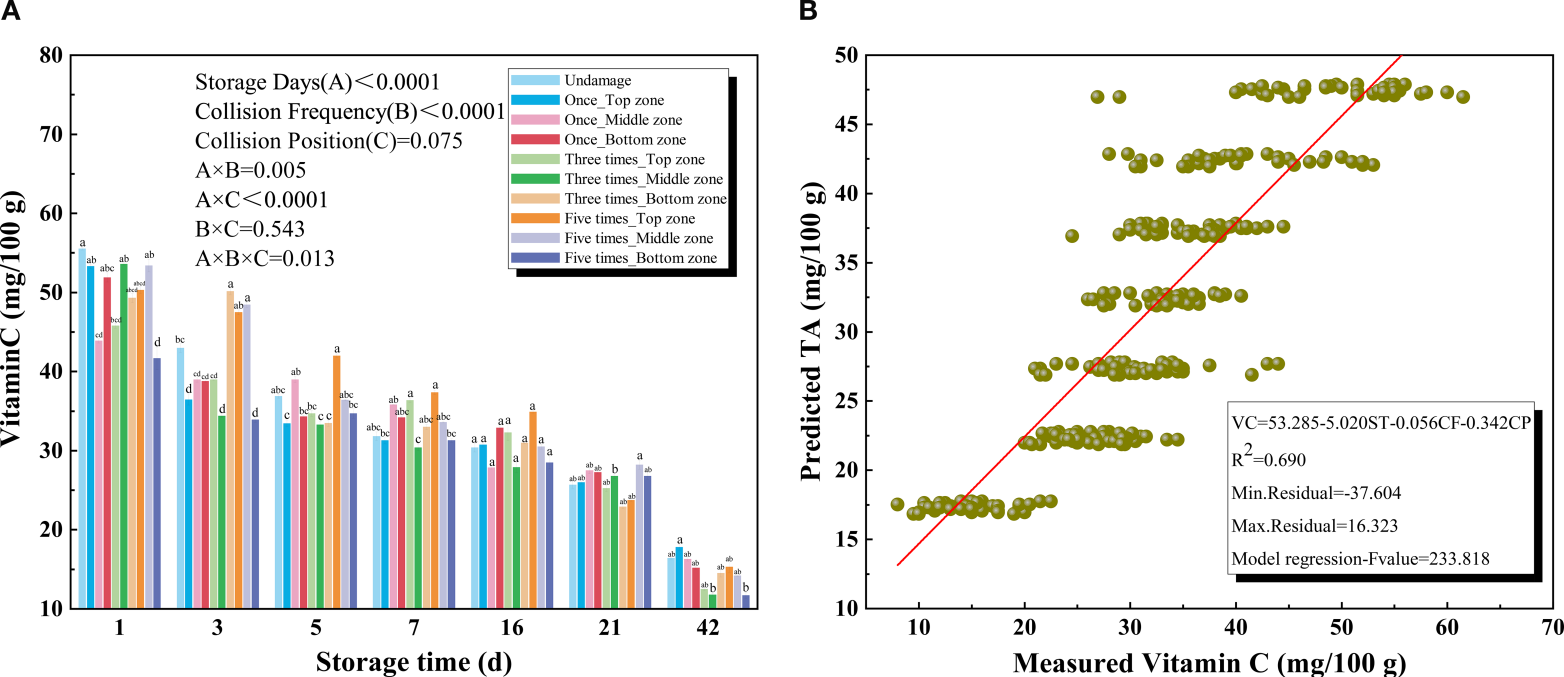
**(A)** Vitamin C (VC) of different collision positions and frequency over 42 days **(B)** Results for the prediction models of vitamin C (VC) based on multiple regression models.

As shown in **Figure 7A**, the vitamin C (VC) content of kiwifruit decreased significantly during the storage period. During the initial 1–7 days, the VC content in kiwifruit remains relatively high, whereas a pronounced decline occurred during the later stage of storage (16–42 days) under all treatment conditions. The storage duration had a significant effect on the VC content of kiwifruit. Furthermore, increased impact frequency accelerated VC degradation. Analysis of variance revealed a significant relationship between collision frequency and VC content (P < 0.0001), whereas collision position had no significant effect (P = 0.075). But, among fruit zones, the top region was most effective in preserving VC, particularly under repeated impacts, while the bottom zone was most susceptible, exhibiting the lowest VC content across all impact scenarios.

In the early stages of storage, exposure to oxygen promotes the oxidation of VC, leading to rapid degradation (Tavarini et al., 2008). Additionally, it has been reported that as fruits become overripe, VC levels decline in parallel with tissue breakdown (Kalt, 2005). Consequently, VC content consistently decreases throughout storage as kiwifruit ripens. Repeated collisions could exacerbate kiwifruit damage by breaking the skin and cell walls, thereby increasing exposure to oxygen and further influencing VC content. **Figure 7B** presented the results of the VC model and all independent variables. The coefficient of determination (R^2^) was 0.690 between the predicted and measured values of VC.

### 3.3 Correlation analysis between the main indicators

**Figure 8:**
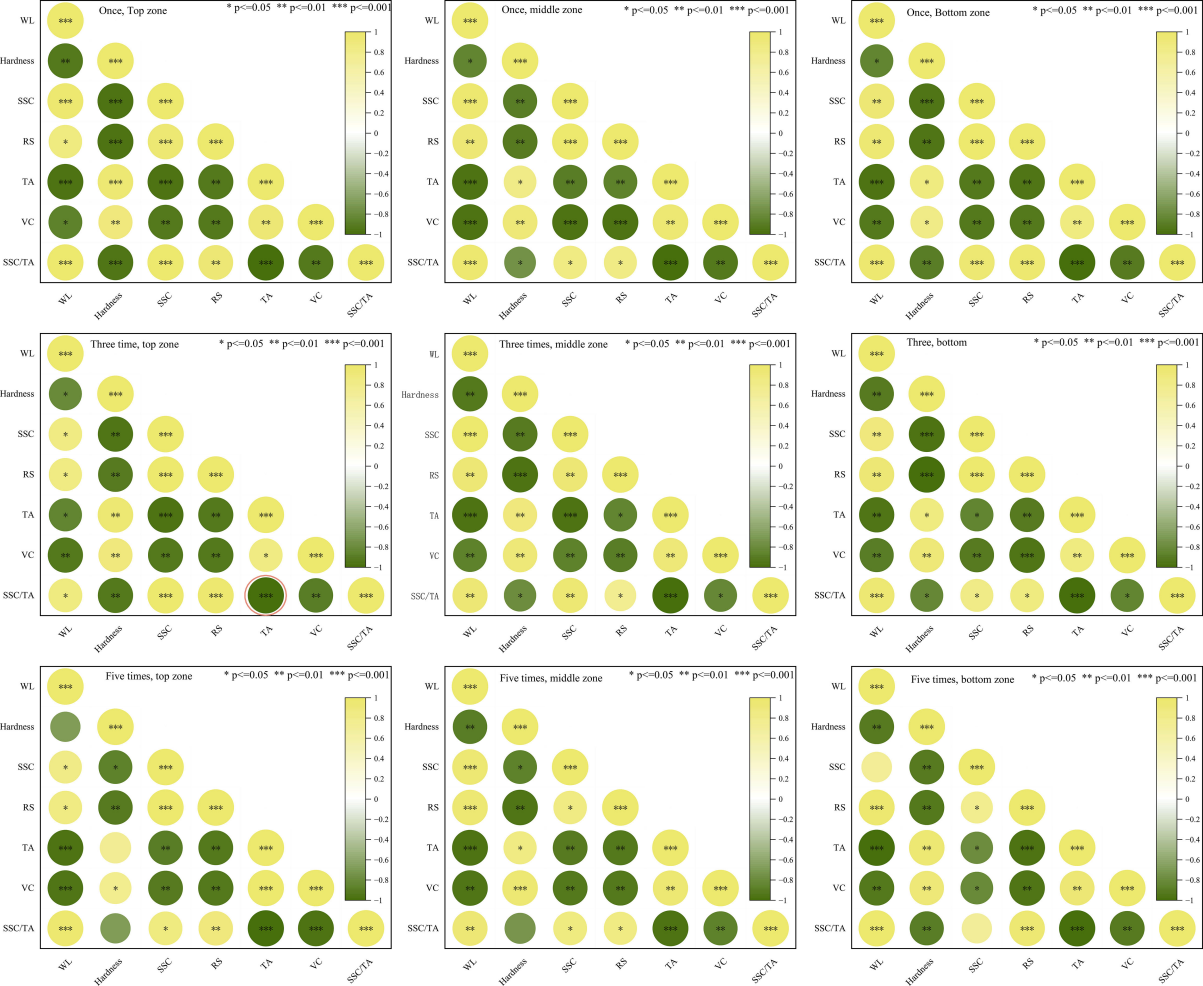
Heatmap of Pearson correlation coefficient (r) matrix between weight loss (WL), hardness, soluble solids content (SSC), reducing sugar (RS), tithable acidity (TA), vitamin C (VC), maturity index (SSC/TA). Top zone, stem shoulder; middle zone, cheek zone; bottom zone, calyx shoulder). The yellower and greener the color, the more correlation indicated. The color range of the heat map cells from green ((high correlation, r = -1) to yellow (high anticorrelation, r = 1), passing from white (no correlation, r = 0). Significance level was set to 0.05, 0.01, 0.001.

The Pearson correlation coefficient matrix is shown as a heat map (**Figure 8**). The Pearson correlation coefficient is used to provide the linear correlation between physicochemical properties of kiwifruits, which is subjected to repeated impact series (once, three times, five times) at three fruit surface zones (stem shoulder, cheek zone, calyx shoulder). The color range of the heat map cells is from light yellow (high correlation, r=1) to green (high anti-correlation, r=-1), passing from white (no correlation, r = 0). Regarding the correlation between chemical quality attributes, there was a significant negative correlation between the WL with hardness, TA, and vitamin C of kiwifruits in all tested conditions (r >- 0.926). There was a significant negative correlation between the WL and SSC, RS of kiwifruits for all tested conditions (r > 0.958). The hardness showed a significant correlation with SSC, RS, and vitamin C content (r > 0.905). A positive correlation was observed between RS with SSC in all kiwifruit fruits across all tested conditions, and a negative correlation was observed between RS with TA and vitamin C content. Meanwhile, vitamin C content displayed a negative correlation with RS. Generally, repeated impact on the stem shoulder and cheek zone of kiwifruit can reduce the correlation between hardness and other physicochemical properties, especially WL, TA. Repeated impact on the fruit at its calyx shoulder reduced the correlation between the SSC of the fruit. The correlation between the physical and physicochemical properties of kiwifruit is not significantly affected by slight impacts (Famiani et al., 2012; Cheng et al., 2022).

## 4 Conclusion

This study investigated the effects of repeated collisions on the physicochemical properties of kiwifruit, revealing that impacts at different surface zones influence fruit quality during storage. Repeated collisions notably increased weight loss and accelerated the reduction in firmness, particularly when applied to the calyx shoulder, which was identified as the most susceptible area to mechanical stress. Soluble solids content (SSC) and reducing sugar (RS) levels generally increased with storage time. Both the frequency and position of collisions exerted significant effects on the contents of SSC and RS. Titratable acidity (TA) and vitamin C (VC) content significantly decreased over time. An increase in collision frequency did not significantly affect TA content but had a significant impact on VC content. After collision damage at the calyx shoulder, long term storage significantly affected TA content, whereas damage at different positions did not significantly influence changes in VC content. Furthermore, repeated collisions weakened the correlations among various physicochemical parameters. These findings underscore the importance of minimizing mechanical impacts, particularly to the cheek and calyx shoulder regions, during postharvest handling and storage to preserve the sensory and nutritional quality of kiwifruit. This study enhances the understanding of collision-induced damage in kiwifruit and offers new insights for improving packaging and processing strategies.

## Data Availability

The original contributions presented in the study are included in the article/supplementary material. Further inquiries can be directed to the corresponding authors.
